# The Small Rho GTPases Rac1 and Rac2 Are Important for T-Cell Independent Antigen Responses and for Suppressing Switching to IgG2b in Mice

**DOI:** 10.3389/fimmu.2017.01264

**Published:** 2017-10-06

**Authors:** Natalija Gerasimčik, Minghui He, Carin I. M. Dahlberg, Nikolai V. Kuznetsov, Eva Severinson, Lisa S. Westerberg

**Affiliations:** ^1^Department of Molecular Biosciences, The Wenner-Gren Institute, Stockholm University, Stockholm, Sweden; ^2^Department of Rheumatology and Inflammation Research, Institute of Medicine, University of Gothenburg, Gothenburg, Sweden; ^3^Department of Microbiology Tumor and Cell Biology, Karolinska Institutet, Stockholm, Sweden; ^4^Department of Medicine Huddinge, Karolinska Institutet, Stockholm, Sweden

**Keywords:** B cells, Rac1, Rac2, Ig class switching, humoral immune response

## Abstract

The Rho GTPases Cdc42, Rac1, and Rac2 coordinate receptor signaling to cell adhesion, migration, and proliferation. Deletion of Rac1 and Rac2 early during B cell development leads to failure in B cell entry into the splenic white pulp. Here, we sought to understand the role of Rac1 and Rac2 in B cell functionality and during the humoral antibody response. To circumvent the migratory deficiency of B cells lacking both Rac1 and Rac2, we took the approach to inducibly delete Rac1 in Rac2^−/−^ B cells in the spleen (Rac1^B^Rac2^−/−^ B cells). Rac1^B^Rac2^−/−^ mice had normal differentiation of splenic B cell populations, except for a reduction in marginal zone B cells. Rac1^B^Rac2^−/−^ B cells showed normal spreading response on antibody-coated layers, while both Rac2^−/−^ and Rac1^B^Rac2^−/−^ B cells had reduced homotypic adhesion and decreased proliferative response when compared to wild-type B cells. Upon challenge with the T-cell-independent antigen TNP-conjugated lipopolysaccharide, Rac1^B^Rac2^−/−^ mice showed reduced antibody response. In contrast, in response to the T-cell-dependent antigen sheep red blood cells, Rac1^B^Rac2^−/−^ mice had increased serum titers of IgG1 and IgG2b. During *in vitro* Ig class switching, Rac1^B^Rac2^−/−^ B cells had elevated germline γ2b transcripts leading to increased Ig class switching to IgG2b. Our data suggest that Rac1 and Rac2 serve an important role in regulation of the B cell humoral immune response and in suppressing Ig class switching to IgG2b.

## Introduction

The Rho GTPases are important for actin cytoskeletal regulation, cell survival, and proliferation (1). The role of one member, Cdc42, on B cell differentiation and function has been studied by us and others. These studies show that Cdc42 is important for B cell differentiation, signal transduction through different receptors, and for the humoral immune response (2–4). Absence of Cdc42 during differentiation in the bone marrow causes an early block in B cell development. B cells devoid of Cdc42 have decreased migration, are unable to polarize the cell body, and have reduced spreading on lipid bilayers membranes (3). Moreover, Cdc42-deficient B cells are impaired in B cell receptor signaling, are unable to mount an antibody response, and have decreased generation of plasma cells (PC) (2, 3). Similar to Guo et al., we found that the migratory response to the chemokine CXCL12 was similar in Cdc42-deficient and wild-type (WT) B cells (2, 4). When examining cytoskeletal responses, B cells lacking Cdc42 had reduced spreading capacity on antibody-coated surfaces, a response coupled to a particular movement of B cells with formation of long trailing uropods (4).

The Rho GTPase Rac1 and its close homolog Rac2 regulate specific responses in B cells. Using ICAM-coated lipid bilayers, Arana et al. showed that B cells lacking Rac2 had reduced B cell receptor-dependent clustering and synapse formation (5). Henderson et al. showed that B cells lacking Rac2 only, or lacking both Rac1 and Rac2, were severely compromised in responses to chemokines and Rac1^−/−^Rac2^−/−^ B cells failed to enter into the splenic white pulp (6). We recently showed that B cells stimulated with anti-CD40 + IL-4 have a more than 80-fold and 120-fold upregulation of mRNA transcripts encoding Rac1 and Rac2, respectively, when compared to non-stimulated cells (7). We have previously shown that anti-CD40 + IL-4 stimulation of B cells leads to formation of microvilli in cell-to-cell contacts (8, 9). This indicated to us that Rac1 and Rac2 might be involved in B cell activation and important for specific cytoskeletal responses.

The role of Rac1 and Rac2 in mature B cells is incompletely understood. Here, we investigated the role of Rac1 and Rac2 in B cell functionality and the humoral immune response. To overcome the homing defect of immature B cells devoid of Rac1 and Rac2 (6), we took the approach to inducibly delete Rac1 in Rac2-deficient B cells (Rac1^B^Rac2^−/−^ B cells). We found that Rac1^B^Rac2^−/−^ B cells had normal capacity to spread on antibody-coated surfaces. In contrast, homotypic adhesion was severely compromised in anti-CD40 + IL-4 stimulated Rac1^B^Rac2^−/−^ B cells. Rac1^B^Rac2^−/−^ mice had increased production of IgG1 and IgG2b in response to T-cell dependent (TD) particulate antigen, but reduced antibody production to T-cell independent (TI) antigen. Upon *in vitro* activation, Rac1^B^Rac2^−/−^ B cells responded with an IgG2b response more than three times higher than WT cells and this was reflected in a significantly elevated level of germline (GL) γ2b transcripts. Together, our data suggest that Rac1 and Rac2 act together to regulate B cell homotypic adhesion, Ig class switching, and the humoral immune response.

## Materials and Methods

### Mice

Rac1^flox^Rac2^−/−^ mice were a kind gift from V. Tybulewicz (MRC National Institute for Medical Research, UK). Mb1-cre-ERT2 mice were a kind gift from M. Reth (University of Freiburg). They were made by inserting Cre-ERT2 into the *Cd79a* locus that encodes Igα [*Cd79a*^Tm3(cre/ESR1)Reth^, *Cd79a*^CreERT2^] (10). All strains were on a C57Bl/6 background, and all mice were bred in specific pathogen-free conditions at the animal facility of the Department of Molecular Biosciences, the Wenner-Gren Institute, Stockholm University. The Rac2^−/−^ and the Rac1^B^Rac2^−/−^ mice were derived from the same littermates. The Rac1^flox^ and wild types were most often from the same littermates, but sometimes the wild types were from a different breeding. However, all breedings pairs were derived from the same recent ancestors, to ensure that background genes would not play a part in the responses. Mice were given tamoxifen (5 mg in 50 µl) by gavage for 5 days in a row to delete Rac1 and sacrificed on days 3 or 4 after the final tamoxifen treatment. For *in vitro* cultures, mice were sacrificed on day 3 after the final tamoxifen treatment. All experiments using mice were approved by a local ethical committee on animal experiments.

### Immunizations

Mice were immunized with TNP-SRBC or TNP-conjugated lipopolysaccharide (TNP-LPS) (Bioresearch Technologies) on day 4 after the final tamoxifen treatment. The erythrocytes were diluted to a 10% mixture from packed cells, and 0.2 ml was injected i.p. TNP-LPS (5 μg/mouse diluted in 0.2 ml) was injected i.p. Mice were bled from the tail or by retro-orbital bleeding in anesthesized mice.

### ELISA

Diluted serum samples were added to 96-well plates, pre-incubated with TNP-BSA, and thereafter washed and incubated with binding buffer. Plates were washed after serum addition, and alkaline phosphatase-conjugated antibodies to IgM or IgG were added and measured by ELISA. Standard monoclonal anti-TNP antibodies were used for the IgM and IgG1 responses. For the IgG2b and IgG3 responses, we used pooled antisera from immunized wild-type (WT) mice in different dilutions as standard, to be able to calculate relative concentrations of the tested sera. An arbitrary value of 100 units corresponded to pooled antisera diluted 1:100 for IgG3. For the IgG2b response, 100 U corresponded to 1:300 dilution after immunization with TNP-LPS and 1:100 after immunization with TNP-SRBC.

### Cell Culture

B cells were purified from spleens by negative selection, using a mouse B cell enrichment kit (Stem Cell Technologies). This method yields >95% pure B cells. For analysis of Ig class switching, spleen B cells were enriched by incubation on ice with antibodies to CD4, CD8, CD90.2, and CD11b (BD Biosciences or eBioscience), thereafter washed and low-tox rabbit complement (Cedarlane) was added. Cells were incubated for 1 h at 37°C and then separated in a Percoll gradient (GE Healthcare). The antibody and complement method yields around 80% B cells. Ig class switching responses are higher using this method, most likely because stromal cells are needed for optimal responses. B cells were cultured at 2–4 × 10^5^ cells/ml as previously described (9). Monoclonal rat anti-mouse CD40 (1C10) was purified as previously described (11) and was used at 10–20 µg/ml. Lipopolysaccharide (LPS) O55:B5 purified by phenol extraction (Sigma-Aldrich) was used at 10 µg/ml. IL-4, IL-5, TGFβ, BAFF, April, and IFNγ, were purchased from Peprotech. IL-4 was used at 8–16 ng/ml, IL-5 was used at 5 ng/ml, TGFβ was used at 0.5 ng/ml, IFNγ was used at 30 ng/ml, and BAFF and APRIL were used at 100 ng/ml. F(ab′)_2_ goat-anti-mouse IgM (Jackson Immunoresearch) was used at 2 µg/ml. Cell spreading assays were performed on glass coverslips coated with anti-CD44 antibodies (BD Biosciences) in 1 ml cultures, as described (8). Spread cells were defined as cells with at least one protrusion longer than one cell diameter. Homotypic aggregation and re-aggregation was performed as described (12).

### DNA Synthesis

Purified B cells were cultured at 10^6^ cells/ml in 0.2 ml in 96-well cultures in complete RPMI1640 plus 10% FCS. [^3^H]thymidine (5 μCi/culture, 20 Ci/mmol, PerkinElmer) was added 17 h before harvesting. Cultures were harvested and incorporated radioactivity was measured using a Wallac microplate scintillation counter (Wallac Oy, Turku, Finland).

### Flow Cytometry

Single-cell suspensions were labeled with fluorescently conjugated anti-mouse antibodies, including anti-B220, -CD21, -CD23, -CD38, -CD86, -CD95 (Fas), -CD138 (Syndecan), -GL7, -CXCR4, -IgM, -IgD, and -IgG2b (BD Biosciences, eBiosciences, Biolegend). A LIVE/DEAD Fixable Dead Cell Stain Kit (Molecular Probes) was used to identify viable cells. Different spleen B cell populations were defined as following: T1 (B220^+^CD23^−^IgM^+^CD21^−/lo^), T2-MZP (B220^+^CD23^+^IgM^+/hi^CD21^+/hi^), FoB (B220^+^CD23^+^IgM^+/lo^CD21^+/lo^), marginal zone B cells (MZB) (B220^+^CD23^−^IgM^+^CD21^hi^), germinal center (GC) B cells (B220^+^IgD^−^GL7^+^CD95^+^), GC light zone (LZ) and dark zone (DZ) B cells (B220^+^IgD^−^GL7^+^CD95^+^CD86^+/−^CXCR4^+/−^), and PC (CD38^−^B220^−^CD138^+^). Fluorescence minus one controls were used when defining different populations of B cells and isotype controls were used in switching experiments. For Ig class switching, single-cell suspensions were fixed with formaldehyde, permeabilized with saponin and labeled with biotinylated antibodies to IgM, IgG1, IgG2b, IgG3, IgE, IgA, or isotype controls (BD Biosciences). Streptavidin-FITC was used as a second step. Data were acquired on FACSCalibur, FACSVerse, or LSR Fortessa (Becton Dickenson). Analyses were made using FlowJo (TreeStar, Inc.).

### Live Cell Imaging

Cell spreading and aggregation were analyzed using a ZEISS Axiovert 200M Cell Observer and SlideBook 5.0 Software. A HAL100 halogen lamp was used for bright-field imaging. Images were taken with an AxioCam MRc digital camera. Two lenses were used: an EC-Plan-Neofluar 10×/0.3 Ph1 and an EC-Plan-Neofluar 40×/0.75 Ph2. Experiments were performed at 37°C in medium supplemented with 10 mM HEPES buffer to keep constant pH.

### Immunofluorescence Microscopy

B cells were activated for 20 h with anti-CD40 + IL-4, transferred to anti-CD44-coated glass cover slips and incubated for an additional 22 h. They were subsequently fixed, washed, permeabilized, treated with anti-mouse CD16/CD32, and incubated with rat anti-mouse α-tubulin antibodies (Abcam). After washing, cells were further stained with fluorescently conjugated donkey anti-rat Ig (which had minimal cross-reactivity with mouse Ig; Jackson ImmunoResearch), fluorescently conjugated phalloidin (Sigma-Aldrich) and Hoechst 33258 (Sigma-Aldrich). Cells were observed and images were captured using a Leica DMLB Fluorescent Microscope, with an HCX PL APO 63×/1.32 oil lens, DC350F CCD camera, and IM500 Software (Leica Microsystems).

### Western Blot

Non-stimulated and stimulated B cells were harvested, and 20 µg of total cell extracts were separated by SDS-PAGE. Western blotting was performed using the Bolt electrophoresis system (Invitrogen) and Bolt™ 10% Bis-Tris Plus polyacrylamide gels (Thermo Fisher Scientific). Antibodies were used as follows: Rac1 (C-14, sc-217, Santa Cruz Biotechnology), Rac2 (ab2244, Abcam), and GAPDH (FL-355, Santa Cruz Biotechnology). Detection of GAPDH was used as a sample loading control. Blots were developed using IgG-HRP labeled antibodies (Santa Cruz Biotechnology) and the ECL detection system. Blot images were analyzed using the ImageQuant LAS 4000 Image system and Control Software (GE Healthcare). The digital image of pre-stained Protein Standard SeeBlue Plus2 (Thermo Fisher Scientific) derived from the same gel run, was overlayed onto chemiluminescent blot image using Adobe Photoshop CS6. Quantification of the bands was performed using ImageJ software (NIH) and relative density was obtained by dividing intensities of Rac1 and Rac2 to intensities of GAPDH. The result was normalized to the relative densities of WT.

### Statistical Analysis

Statistically significant differences between groups were assessed by Student’s *t-*test for paired or unpaired analysis and ordinary one-way or two-way ANOVA with Sidak’s or Tukey’s tests for multiple comparisons. Differences were considered significant when *p* ≤ 0.05.

## Results

### Deletion of Rac1 in Rac2^−/−^ B Cells Leads to Normal Differentiation of Follicular B Cells But Reduced MZB

**Figure 1:**
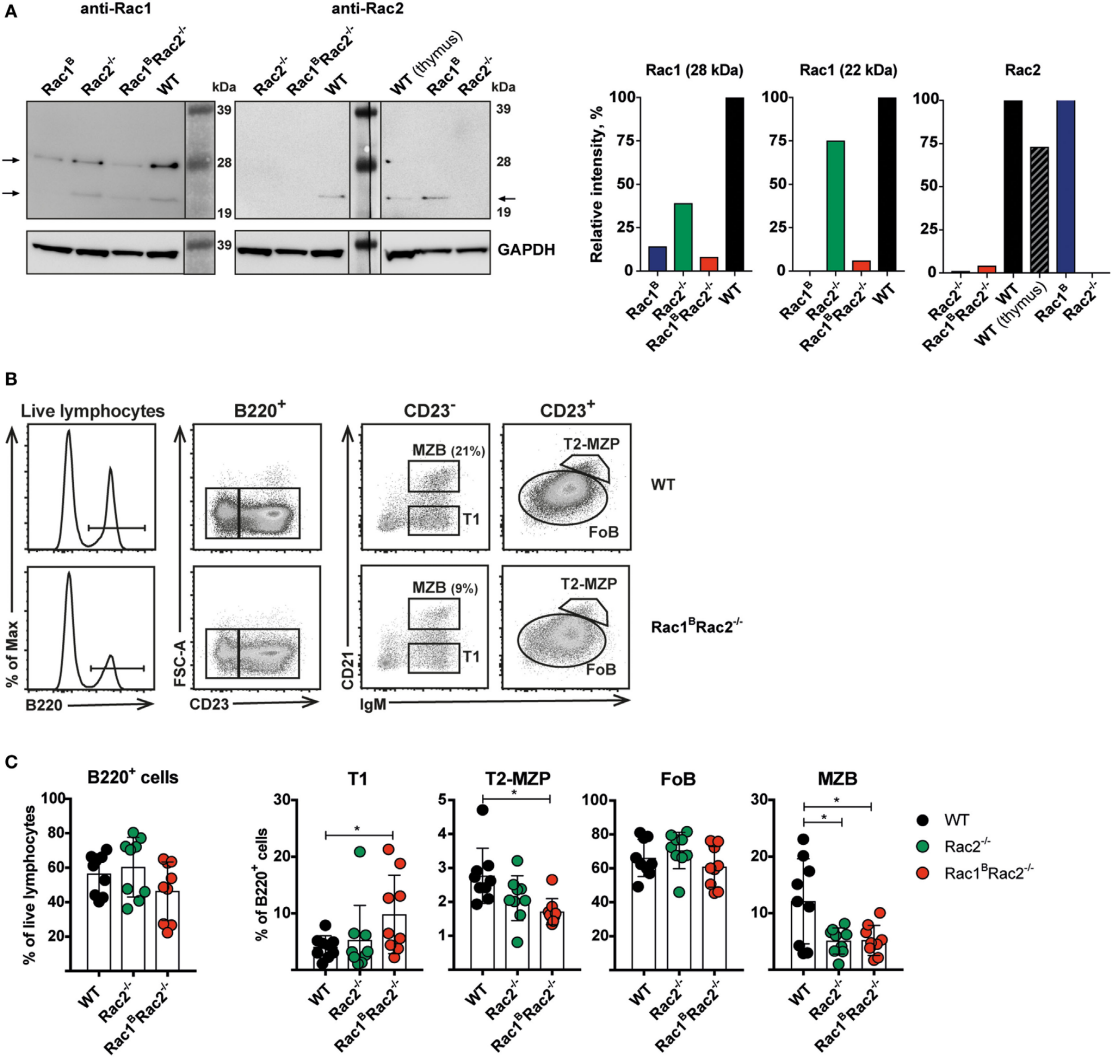
Deletion of Rac1 in Rac2^−/−^ B cells leads to normal differentiation of follicular B cells but reduced marginal zone B cells (MZB). **(A)** Western blots (left) show the efficiency of Rac1 and Rac2 protein deletion in resting B cells, 3 days after the last tamoxifen-injection. Graphs (right) show the intensities of the Rac1 and Rac2 bands relative to those of GAPDH and normalized to the wild-type (WT) bands. **(B)** Gating strategy for T1, T2-MZP, FOB, and MZB cells in representative WT and Rac1^B^Rac2^−/−^ B cells. **(C)** Percentage of B cells (B220^+^), T1, T2-MZP, FOB, and MZB cells. Each dot represents one mouse and *n* = 9. Analysis was performed 13 days after the last tamoxifen-injection. Statistical analysis done using unpaired two-tailed *t*-test. *p < 0.05.

To investigate how deletion of Rac1 and Rac2 would affect B cell functionality and the GC response, we took the approach to inducibly delete Rac1 in Rac2^−/−^ B cells already residing in the spleen. We bred Rac2^−/−^ mice with mice bearing a conditionally targeted Rac1 allele (Rac1^flox^) and Mb1-Cre-ERT2, referred to as Rac1^B^Rac2^−/−^ mice. In WT spleen B cells, we detected two molecular weight species of Rac1 corresponding to the 22 and 28 kDa. For Rac2, we detected one molecular species corresponding to the 22 kDa splice forms. Quantification of the bands revealed that the Rac1 antibody cross-reacted somewhat with Rac2, and this was especially evident for the 28 kDa band. The Rac2 antibody seemed less crossreactive. Upon tamoxifen administration for five consecutive days, Rac1 was efficiently deleted in Rac1^B^ and Rac1^B^Rac2^−/−^ resting B cells on day 8 (Figure 1A). We next examined B cell populations in the spleen. WT, Rac2^−/−^, and Rac1^B^Rac2^−/−^ mice had similar percentages of total B220^+^ B cells and follicular B cells (FOB) (Figures 1B–C). Transitional type 1 (T1) B cells were increased in Rac1^B^Rac2^−/−^ mice, whereas transitional type 2 marginal zone precursors (T2-MZP) were lower when compared to WT and Rac2^−/−^ mice (Figure 1C). Both Rac2^−/−^ and Rac1^B^Rac2^−/−^ mice showed lower percentage of MZB (Figure 1C).

### Normal B Cell Spreading and Reduced Homotypic Adhesion by Rac2^−/−^ and Rac1^B^Rac2^−/−^ B Cells

**Figure 2:**
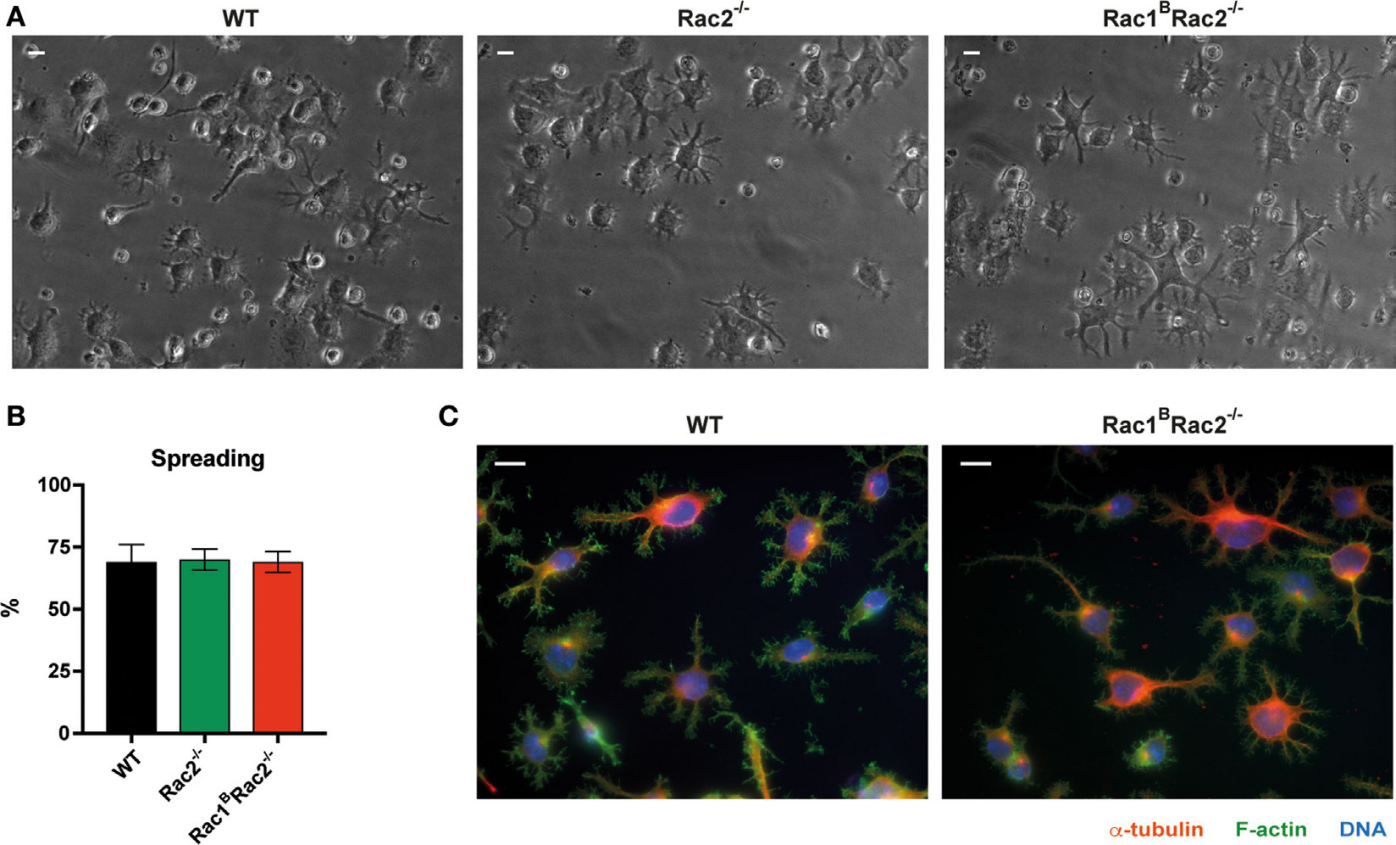
Normal spreading response by Rac1^B^Rac2^−/−^ B cells. **(A)** Bright-field images of spread B cells, magnification 40×. Scale bar 10 µm. **(B)** Graph showing quantification of the spreading. *n* = 300–400 cells per mouse per experiment. **(C)** Spread cells stained for α-tubulin, (red), filamentous actin (green) and DNA (blue), magnification 63×. Scale bar 10 µm. Mice were sacrificed 3 days after the last tamoxifen-injection. Purified B cells were cultured with anti-CD40 + IL-4 for 2 days. Experiments were repeated 2–3 times with similar results.

**Figure 3:**
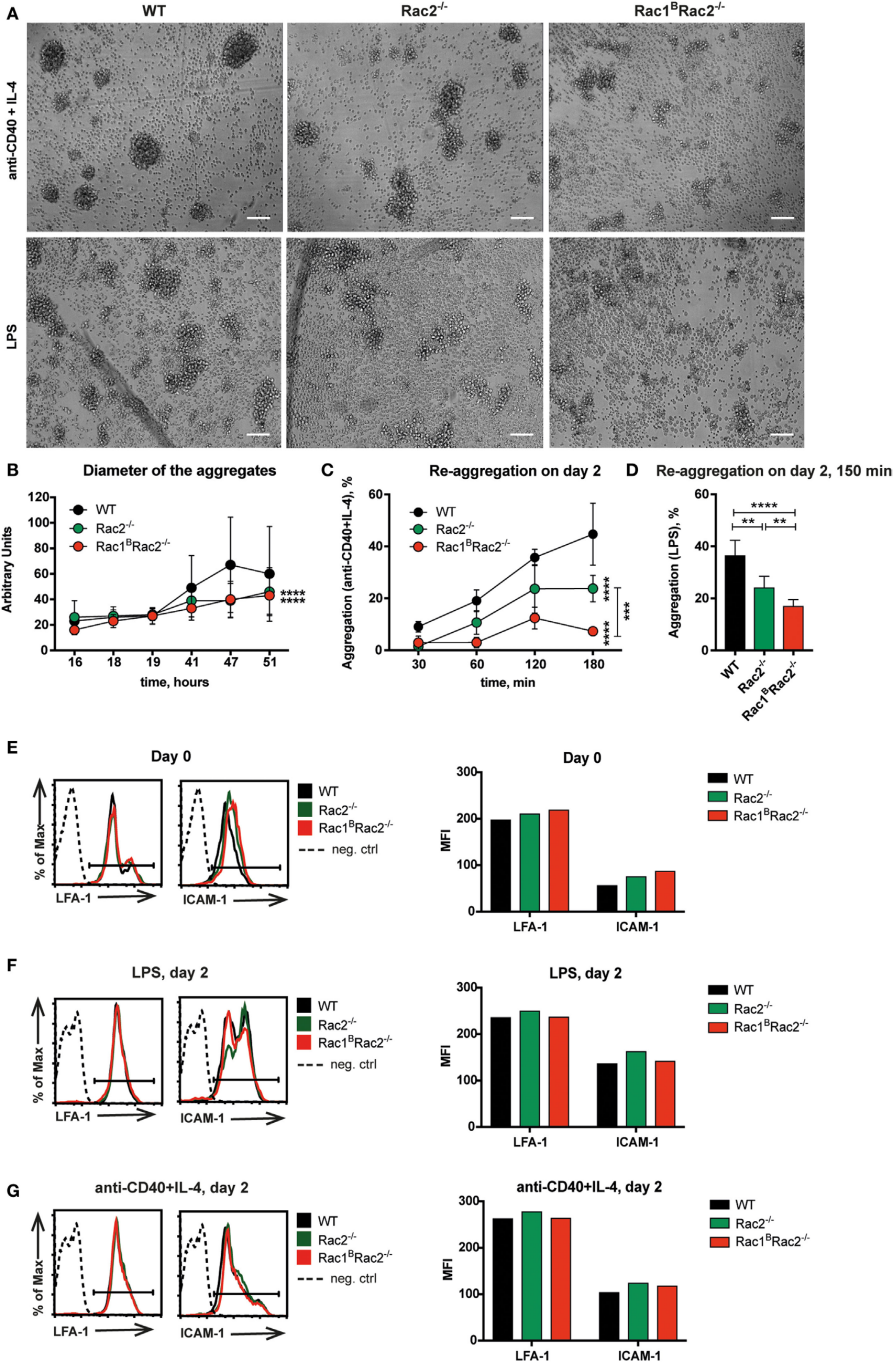
Reduced homotypic aggregation in Rac1^B^Rac2^−/−^ B cells. Purified B cells were cultured 3 days after the last tamoxifen-injection. **(A)** Cells were activated for 2 days with anti-CD40 + IL-4 (top) or lipopolysaccharide (LPS) (bottom). Representative live cell imaging photos show aggregation of B cells, magnification 10×. Scale bars 100 µm. **(B)** Diameter of the aggregates after stimulation with anti-CD40 + IL-4 for indicated times. **(C,D)** Quantification of re-aggregation. Cells were stimulated for 2 days with anti-CD40 + IL-4 **(C)** or LPS **(D)**, then dissociated to single cells by pipetting, and plated onto black glass-bottom 96-well plates. The percentage of aggregates was determined at the indicated time points thereafter. **(E–G)** LFA-1 and ICAM-1 expression on day 0 on B220^+^ cells **(E)**, on day 2 after LPS stimulation **(F)**, and on day 2 after stimulation with anti-CD40 + IL-4 **(G)**. **(B)** The experiment was repeated three times with similar results. **(C, D)** The experiment was done in triplicates and repeated once with similar results. **(B,C)** Statistical analysis performed using two-way ANOVA with Sidak’s and Tukey’s tests for multiple comparisons. ***p < 0.001, ****p < 0.0001. **(D)** Statistical analysis performed using unpaired two-tailed *t*-test. **p < 0.01, ****p < 0.0001. **(E–G)** One mouse used per group. The experiment was repeated twice with similar results.

Wild-type B cells stimulated by anti-CD40 + IL-4 show homotypic adhesion with microvilli in cell-to-cell contacts (8, 9). When cultured on antibody-coated monolayers, anti-CD40 + IL-4 stimulated B cells form long thin and branched protrusions characterized as spread B cells (8, 13). We previously demonstrated that Cdc42-deficient B cells have normal homotypic B cell adhesion but almost abolished spreading response (4). Rac1 and Rac2 were dispensable for B cell spreading since WT, Rac2^−/−^, and Rac1^B^Rac2^−/−^ B cells showed similar formation of long protrusions (Figures 2A–C). To understand the role of Rac1 and Rac2 in homotypic adhesion, we examined aggregate formation after activation with anti-CD40 + IL-4 or LPS for 2 days. WT B cells formed round tight aggregates after stimulation with anti-CD40 + IL-4, whereas the aggregates were more loose in character and have uneven borders after activation with LPS (Figure 3A). WT and Rac1^B^ B cells showed similar formation of aggregates induced by anti-CD40 + IL-4 (Figure S1 in Supplementary Material). Rac2^−/−^ and Rac1^B^Rac2^−/−^ B cells formed smaller aggregates and those present in the B cell cultures had irregular shapes (Figures 3A,B). When compared to WT B cells and Rac1^B^ B cells, Rac2^−/−^, and Rac1^B^Rac2^−/−^ B cells had reduced capacity to re-aggregate from single cells on day 2 of anti-CD40 + IL-4 stimulation (Figure 3C). Similarly, LPS activated Rac1^B^Rac2^−/−^ B cells responded by reduced capacity to re-aggregate and Rac2^−/−^ B cells gave an intermediate response (Figure 3D). The reduced formation of homotypic B cell aggregates in Rac2^−/−^ and Rac1^B^Rac2^−/−^ B cells was not caused by lower expression of the main adhesion molecules for this type of response, LFA-1 and ICAM-1, on either naïve B cells or activated B cells (Figures 3E–G). Together, this data suggest that Cdc42 and the Rac proteins regulate different cytoskeletal responses in B cells. We conclude that whereas B cell spreading is controlled by Cdc42, homotypic adhesion is controlled by Rac1 and Rac2.

### Reduced IgM-Induced DNA Synthesis by Rac2^−/−^ and Rac1^B^Rac2^−/−^ B Cells

**Figure 4:**
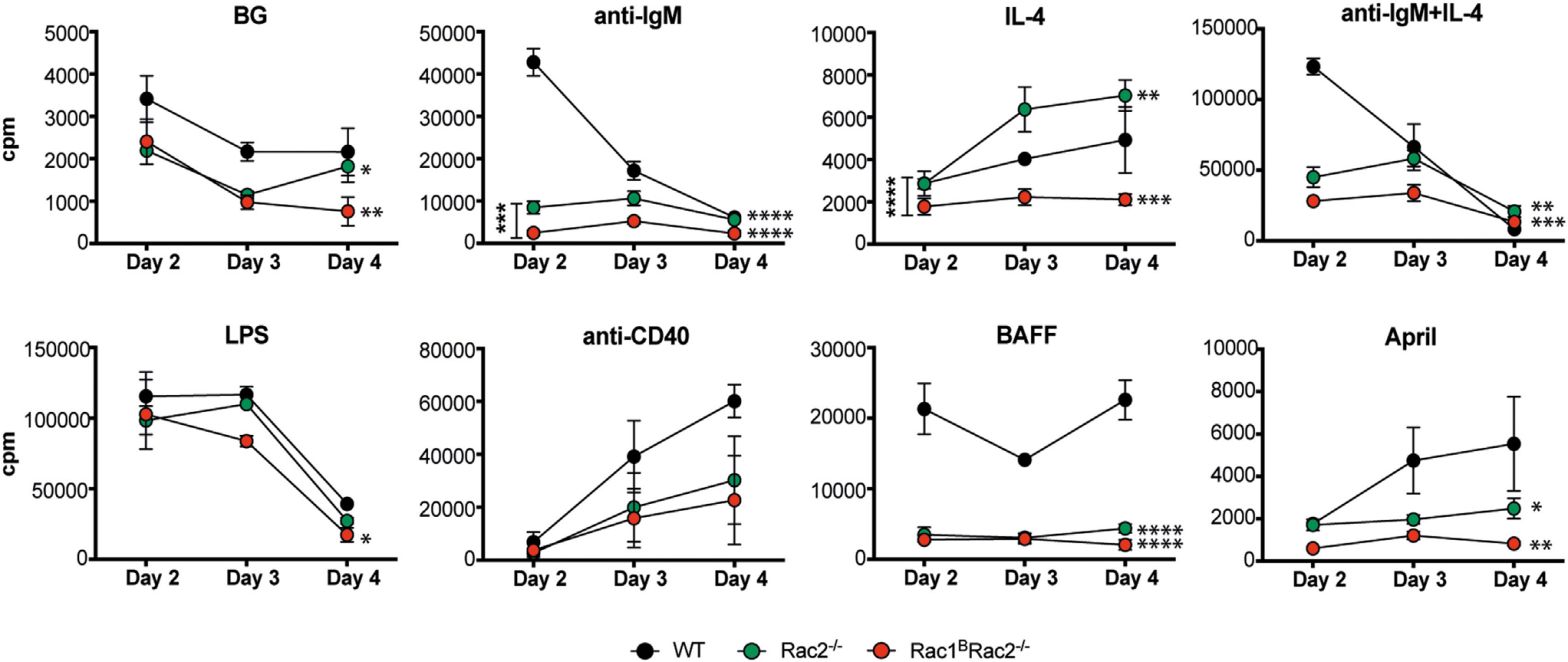
Reduced DNA synthesis by Rac2^−/−^ and Rac1^B^Rac2^−/−^ B cells. DNA synthesis was measured by [^3^H]thymidine uptake of spleen B cells, cultured for 2, 3, and 4 days. Mice were sacrificed 3 days after the last tamoxifen-injection. [^3^H]thymidine was added for the final 17 h. The values represent the mean of triplicates and bars represent SD. The experiment was repeated at least once with similar results. BG, background. Statistical analysis was done using two-way ANOVA with Tukey’s test for multiple comparisons that includes all time points. *p < 0.05, **p < 0.01, ***p < 0.001, ****p < 0.0001.

To address how Rac1 and Rac2 in B cells may be important for signaling from different cell surface receptors, we measured DNA synthesis upon specific receptor stimulation. Rac1^B^ B cells responded similarly to WT B cells to all stimuli tested (data not shown) (14). Rac1^B^Rac2^−/−^ B cells had slightly reduced DNA synthesis response to stimulation by LPS when compared to WT B cells (Figure 4). Rac2^−/−^ and Rac1^B^Rac2^−/−^ B cells showed reduced DNA synthesis to stimulation by anti-IgM and anti-IgM + IL-4, and the cells did not respond to BAFF and April (Figure 4). Rac2^−/−^ B cells had higher levels of DNA synthesis as a response to IL-4 in this experiment, but not in another (data not shown). The modest response to IL-4 is just above background levels. In fact, IL-4 alone does not induce proliferation in B cells, but rather decreased apoptosis and this might be the reason for the slightly elevated response (15). We examined if reduced DNA synthesis in Rac2^−/−^ and Rac1^B^Rac2^−/−^ B cells was due to increased cell death but found similar proportion of apoptotic cells among WT, Rac2^−/−^, and Rac1^B^Rac2^−/−^ B cells (Figure S2 in Supplementary Material). These data suggest that Rac2, but not Rac1, is important for B cell *in vitro* DNA synthesis responses and that the response to LPS is independent of Rac1 and Rac2.

### Rac1^B^Rac2^−/−^ Mice Have Increased IgG1 and IgG2b Titers in Response to a Particulate T Cell Dependent Antigen, But Are Severely Immunodeficient to a T Cell Independent Antigen

**Figure 5:**
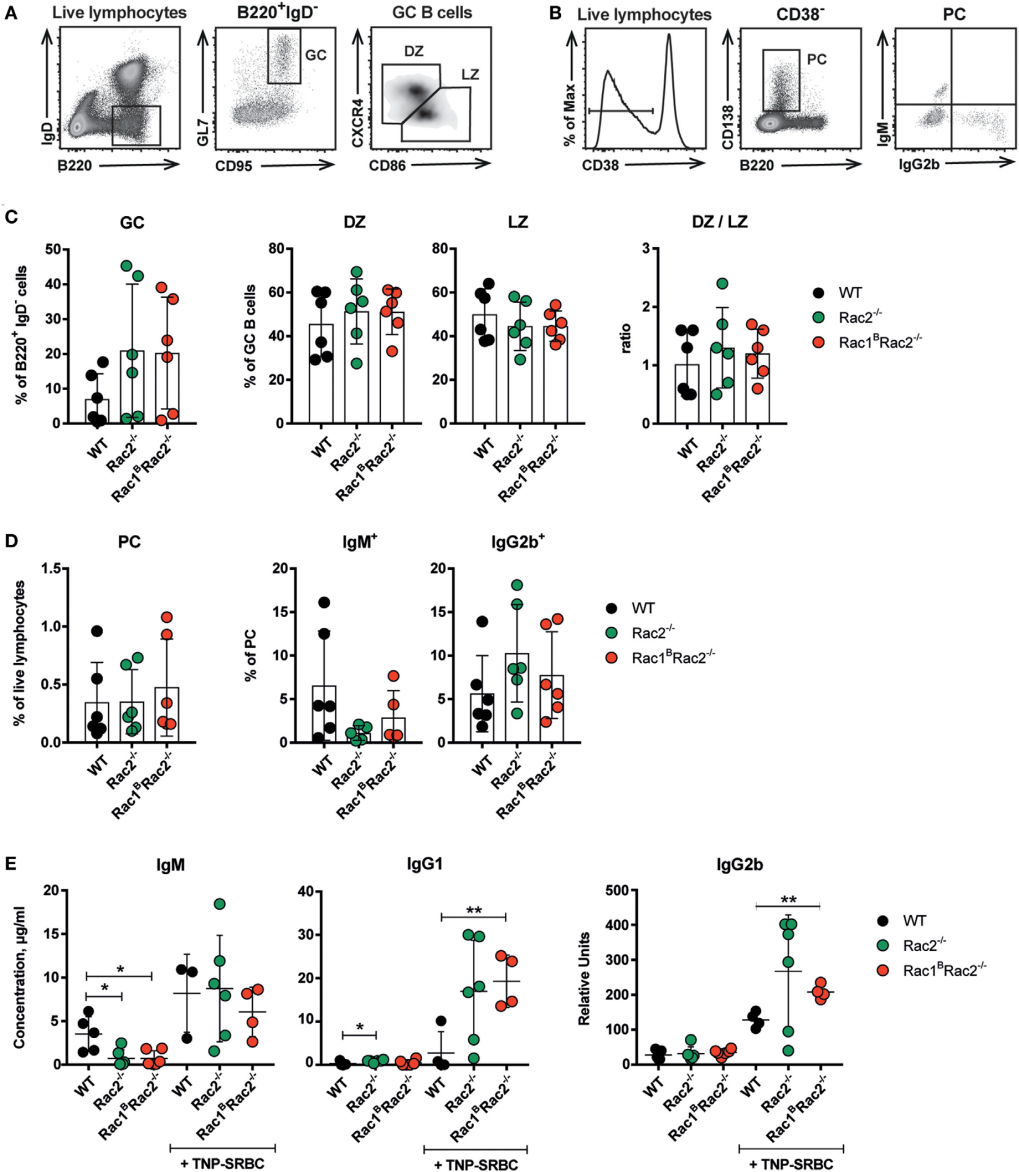
Rac1^B^Rac2^−/−^ mice show increased IgG1 and IgG2b titers in response to TNP-SRBC. **(A,B)** Gating strategy for germinal center (GC), dark zone (DZ), light zone (LZ) **(A)** and total plasma cells (PC), IgM^+^, and IgG2b^+^ PC cells **(B)**. **(C,D)** Percentages of different B cell populations: GC, DZ, LZ, DZ/LZ ratio; total PC, IgM^+^, and IgG2b^+^ PC cells. Each dot represents one mouse. **(E)** IgM, IgG1, and IgG2b response to TNP-SRBC immunization. **(C,D)** *n* = 6 per group, and **(E)** *n* = 4–6 mice per group, where each dot represents one mouse. Mice were sacrificed on day 10 after immunization and on day 13 after the last tamoxifen-injection. Statistical analysis was performed using unpaired two-tailed *t*-test *p < 0.05, **p < 0.01.

**Figure 6:**
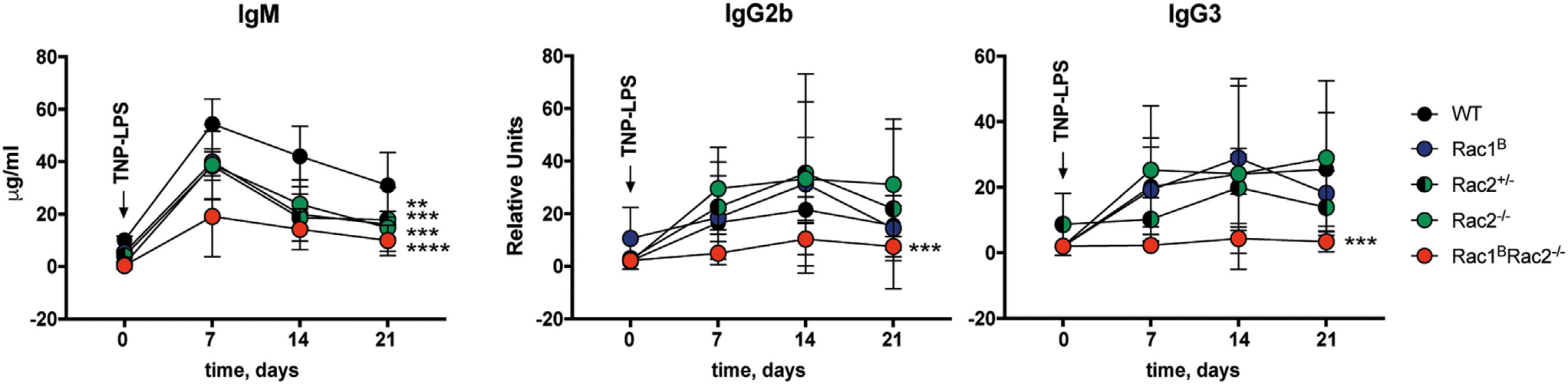
Rac1^B^Rac2^−/−^ mice fail to respond to TNP-conjugated lipopolysaccharide (TNP-LPS). IgM, IgG2b, and IgG3 response to TNP-LPS immunization. Mice were immunized 4 days after the last tamoxifen-injection and bled once a week between day 0 and day 21. Day 0, *n* = 2 mice per group; day 7, *n* = 5 mice per group, except *n*_Rac2+/−_ = 6; day 14, *n* = 5 mice per group, except *n*_WT_ = 3; day 21, *n* = 5 mice per group, except *n*_Rac2+/−_ = 3. Statistical analysis done using two-way ANOVA with Sidak’s and Tukey’s tests for multiple comparisons combining all time points. **p < 0.01, ***p < 0.001, ****p < 0.0001. Statistical comparison for IgM: **Rac1^B^ vs WT, ***Rac2^+/−^ vs WT, ***Rac2^−/−^ vs WT, ****Rac1^B^Rac2^−/−^ vs WT. Statistical comparison for IgG2b and IgG3: ***Rac1^B^Rac2^−/−^ vs WT.

To investigate how deletion of Rac1 and Rac2 in B cells affects the humoral immune response, Rac1^B^Rac2^−/−^ were given tamoxifen followed by immunization with the particulate TD antigen trinitrophenyl-conjugated sheep red blood cells (TNP-SRBC). Immunized mice were sacrificed on day 10 after antigen administration. The percentage of GC B cells, DZ or LZ cells, total PC, and IgM- and IgG2b-producing PC were similar in WT, Rac2^−/−^, and Rac1^B^Rac2^−/−^ mice (Figures 5A–D). These reflect polyclonal responses, which partly might be independent on the immunization. In serum, the IgM response was similar in WT, Rac2^−/−^, and Rac1^B^Rac2^−/−^ mice (Figure 5E), although Rac2^−/−^ and Rac1^B^Rac2^−/−^ mice had lower serum IgM before immunization with TNP-SRBC. Rac1^B^Rac2^−/−^ mice showed increased serum titer of IgG1 and IgG2b when compared to WT mice (Figure 5E). We next tested the response to the TI antigen TNP-LPS. Tamoxifen-injected mice were immunized with TNP-LPS and serum antibody titers were analyzed on days 7, 14, and 21. Rac1^B^, Rac2^+/−^, Rac2^−/−^, and Rac1^B^Rac2^−/−^ mice responded with lower IgM responses when compared to WT mice. Rac1^B^, Rac2^+/−^, and Rac2^−/−^ mice had similar IgG2b and IgG3 responses, whereas Rac1^B^Rac2^−/−^ mice completely lacked IgG2b and IgG3 responses (Figure 6). Together these data suggest that Rac1^B^Rac2^−/−^ mice had severely reduced capacity to induce an antibody response to a TI antigen, but showed increased IgG1 and IgG2b antibody responses to a TD antigen.

### Increased IgG2b Production in Rac2^−/−^ and Rac1^B^Rac2^−/−^ B Cells Stimulated *In Vitro*

**Figure 7:**
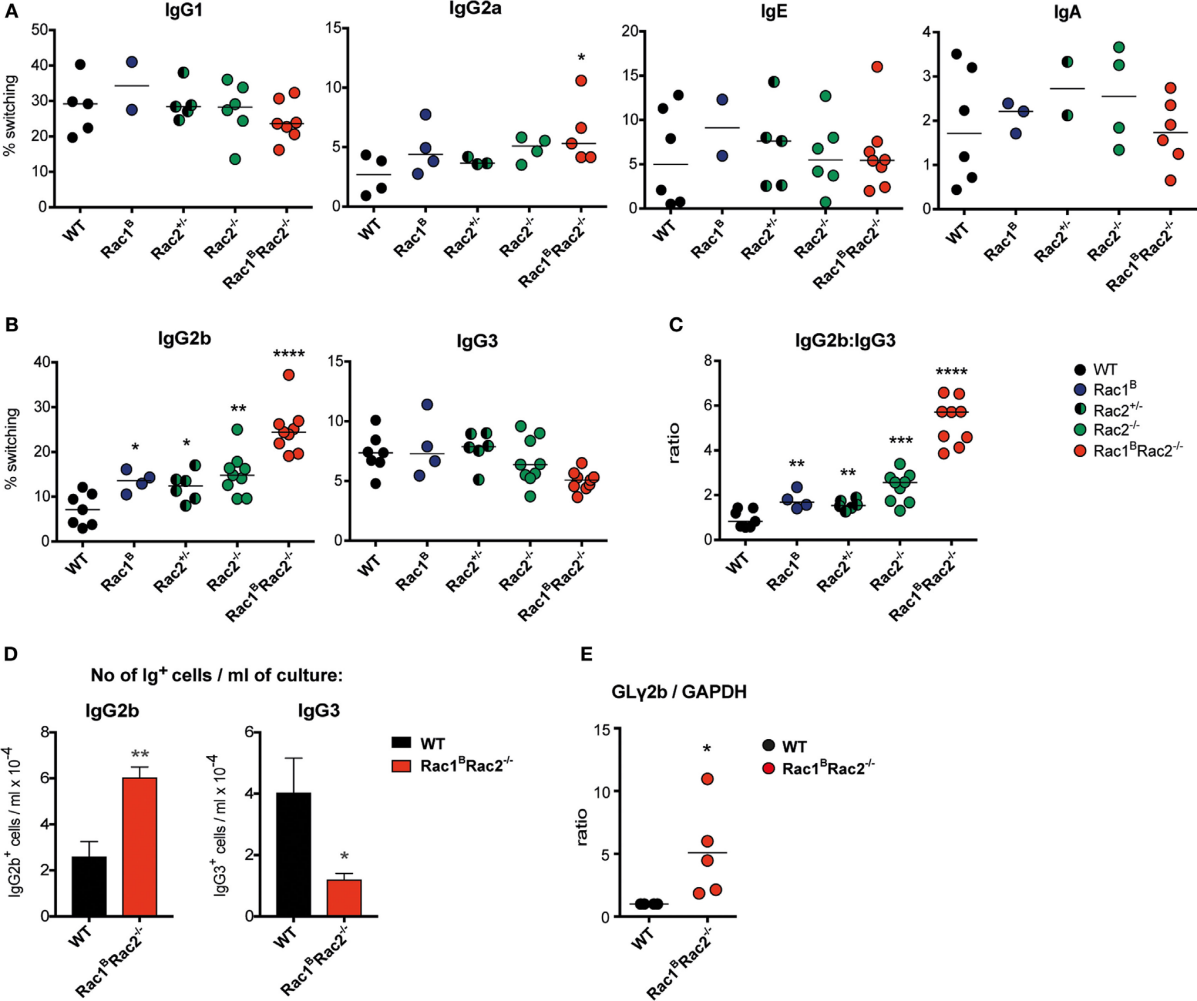
Increased IgG2b production in Rac1^B^Rac2^−/−^ B cells. **(A)** Ig class switching to IgG1, IgG2a, IgE, and IgA. Mice were sacrificed on day 3 after the last tamoxifen-injection and purified B cells were cultured with different stimuli. For IgG1 and IgE, B cells were stimulated with anti-CD40 + IL-4, for IgG2a with lipopolysaccharide (LPS) and IFNγ, and for IgA with LPS + IL-4 + IL-5 + TGFβ for 4 days. *n* = 2–8 mice per group, where each dot represents one mouse. **(B)** Ig class switching to IgG2b and IgG3. B cells were stimulated with LPS for 4 days. *n* = 4–9 mice per group, where each dot represents one mouse. **(C)** Ratio of percentages of IgG2b divided by that of IgG3. Each dot represents one mouse. **(D)** Total number of IgG2b^+^ or IgG3^+^ cells per milliliter of culture after stimulation for 4 days with LPS. Values represent mean of triplicates, bars represent SD. The experiment was repeated once with similar results. **(E)** Germline (GL) γ2b transcripts in wild-type (WT) and in Rac1^B^Rac2^−/−^ B cells after stimulation for 2 days with LPS. The GLγ2b expression was normalized to that of GAPDH. WT was set to 1. Each dot represents one mouse. Statistical analysis was performed using unpaired two-tailed *t*-test **(A–C)**, Student’s non-paired *t*-test **(D)**, and non-parametric Kolmogorov–Smirnov *t*-test (E). *p < 0.05, **p < 0.01, ***p < 0.001, ****p < 0.0001.

To investigate the intrinsic capacity of Rac1^B^Rac2^−/−^ B cells to undergo Ig class switching, we stimulated spleen B cells with cytokines *in vitro* and examined Ig class switching on day 4 (16). WT, Rac1^B^, Rac2^−/−^, and Rac1^B^Rac2^−/−^ B cells had similar switching to IgG1, IgE, and IgA (Figure 7A). When compared to WT B cells, Rac1^B^Rac2^−/−^ B cells had increased IgG2a^+^ B cells in response to LPS + IFNγ (Figure 7A). In response to LPS, Rac1^B^, Rac2^−/−^ and heterozygote Rac2^+/−^ B cells showed higher Ig class switching to IgG2b (Figure 7B). Rac1^B^Rac2^−/−^ B cells had more than threefold higher Ig class switching to IgG2b when compared to WT B cells. In contrast, LPS-induced Ig class switching to IgG3 was similar in WT, Rac1^B^, Rac2^−/−^, Rac2^+/−^, and Rac1^B^Rac2^−/−^ B cells (Figure 7B), leading to a large increase in the IgG2b:IgG3 antibody ratio in Rac1^B^Rac2^−/−^ B cells (Figure 7C). When calculated as numbers of Ig^+^ cells per ml of culture, IgG2b^+^ cells per ml culture was higher whereas IgG3^+^ cells were lower among Rac1^B^Rac2^−/−^ B cells when compared to WT B cells (Figure 7D). We next examined induction of GL γ2b transcripts needed for Ig switching to IgG2b (17). Rac1^B^Rac2^−/−^ B cells had increased GL γ2b when compared to WT B cells (Figure 7E). These data indicate that Rac1 and Rac2 directly or indirectly suppress IgG2b switching by controlling GL γ2b transcription.

## Discussion

We sought to determine the role of Rac1 and Rac2 in B cell functionality *in vitro* and *in vivo*. Considering the pronounced reduction in B cell homing into the splenic white pulp when Rac1 and Rac2 are deleted early during B cell development (6), we found that inducible deletion of Rac1 in Rac2^−/−^ B cells led to normal differentiation of follicular B cells and formation of GC and PC upon antigen challenge with TD antigen. Loss of Rac1 and Rac2 led to altered antibody responses *in vivo*, to decreased receptor-mediated proliferation, and increased Ig class switching to IgG2b *in vitro*. These data suggest that the combined activity of Rac1 and Rac2 in B cells is needed to control the humoral immune response, B cell proliferation, and Ig class switching to IgG2b.

We have chosen to delete Rac1 in an inducible manner, to be able to distinguish the contribution of a deletion at early B cell differentiation stages from that of mature B cells. A consequence is that the phenotype would most likely appear milder than if deletion was made from early differentiation stages. We used a similar strategy in a recent paper to delete Cdc42 in B cells. We found that deletion of Cdc42 was maintained for 2 weeks after the last tamoxifen administration, but Cdc42 expression started to recover by 3 weeks post induced deletion (4).

Cdc42 and Rac1 when microinjected into serum starved fibroblasts induce different cytoskeletal responses. Rac1 induces lamellipodia formation consisting of a highly branched F-actin network, largely mediated by WAVE1–3 proteins (18, 19). Cdc42 induces filopodia formation that contains thick bundles of straight F-actin filaments (20). In light of the different cytoskeletal structures that are induced by Cdc42 and Rac1, it is not surprising that we found different effects on the cell cytoskeleton in B cells lacking Cdc42 when compared to B cells lacking Rac1 and Rac2. We have previously shown that the B cell spreading response induced by anti-CD40 + IL-4 depends on the presence of Cdc42 (7), and we now demonstrate that Rac1 and Rac2 are dispensable for this response. On the other hand, Cdc42 is not required for homotypic B cell adhesion induced by anti-CD40 + IL-4 (7), whereas this response was highly reduced by deletion of both Rac1 and Rac2 and by single deletion of Rac2, as shown in the present paper. This suggests that Rac2, and to a lesser extent Rac1, have unique roles in homotypic B cell adhesion. The adhesion molecules most important for the adhesion responses are ICAM-1 and LFA-1. Upregulation of ICAM-1 occurs after activation of B cells with mitogenic stimuli such as LPS, whereas LFA-1 surface expression remains relatively constant. Instead, LFA-1 binding is controlled by regulating the affinity state (12). Both molecules were expressed equally well after activation in control B cells and those lacking Rac1 and Rac2 or only Rac2. We suggest that Rac1 and Rac2 may be involved in regulation of the high affinity state of LFA-1. The affinity of LFA-1 is regulated directly by its binding to the actin cytoskeleton, possibly *via* activation of the phosphoinositide 3-kinase, and this leads to clustering of LFA-1 molecules on the cell surface (21). However, there has to be a more specific regulation of LFA-1 affinity than mere actin polymerization, since Cdc42 is not involved in this response (4). Since the TD response was not reduced in Rac2^−/−^ and Rac1^B^Rac2^−/−^ mice, the capacity of B cells to adhere to each other seems not to be important for this response. On the other hand, the impaired TI response in these mice might be related to decreased adhesion.

T-cell dependent humoral immune responses are associated with formation of GC (22), whereas TI responses do not induce these structures. Since GC B cells adhere to each other, it is reasonable to assume that homotypic adhesion of B cells would be important for formation of GC. In this paper, we show that GC B cells from Rac1^B^Rac2^−/−^ mice are induced by the TD antigen TNP-SRBC, although the cells showed reduced homotypic adhesion. Interestingly, Cdc42-deficient B cells have reduced spreading and GC responses. Thus, spreading, but not homotypic adhesion of B cells seems important for GC formation.

It is interesting to note that follicular B cells developed normally when Rac1 was inducibly deleted in Rac2^−/−^ B cells, especially since we found that Rac2^−/−^ B cells and to a larger extent Rac1^B^Rac2^−/−^ B cells had much reduced DNA synthesis in response to stimulation with BAFF and April. BAFF is an important survival factor for B cells and in the absence of Rac1 and Rac2, B cells fail to upregulate BAFF receptors on the cell surface (14). Moreover, B cells devoid of Rac1 and Rac2 survive less well when stimulated by anti-IgM antibodies (14). We detected reduced DNA synthesis to anti-IgM stimulation in the presence or absence of IL-4, as well as to stimulation with anti-CD40 in Rac2^−/−^ and Rac1^B^Rac2^−/−^ B cells. However, we found no evidence that Rac2^−/−^ or Rac1^B^Rac2^−/−^ B cells had increased percentage of apoptosis after stimulation with anti-CD40 + IL-4. This suggests that despite the poor response *in vitro* to factors that stimulate follicular B cell proliferation and survival, the *in vivo* context overcomes the reduced responsiveness of Rac1^B^Rac2^−/−^ B cells. In contrast, MZB were highly dependent on Rac2 for normal development from marginal zone precursor B cells.

Analysis of the humoral immune response of Rac1^B^, Rac2^−/−^, and Rac1^B^Rac2^−/−^ mice revealed surprising results. The TD antigen TNP-SRBC induced IgM responses similar in Rac1^B^Rac2^−/−^ mice and controls, whereas the IgG1 and IgG2b responses were elevated in Rac1^B^Rac2^−/−^ mice. On the contrary, Rac1^B^Rac2^−/−^ mice were strongly immunodeficient in their response to the TI antigen TNP-LPS. This reduced response to TNP-LPS may be partly explained by the decreased number of MZB in Rac1^B^Rac2^−/−^ mice. However, this cannot fully explain the results, since Rac2^−/−^ and Rac1^B^Rac2^−/−^ mice are both as deficient in MZB, whereas there is a clear difference in their response to TNP-LPS. Thus, it is likely that there are other, as yet unidentified differences between the Rac2^−/−^ mice and Rac1^B^Rac2^−/−^ mice. Perhaps, more interesting is the increased antibody response to TNP-SRBC in Rac1^B^Rac2^−/−^ mice. Rac1^B^Rac2^−/−^ B cells have poor migratory response to lymphoid chemokines (CXCL12, CXCL13, and CCL21) (6). Our data suggest that deletion of Rac1 in Rac2^−/−^ B cells already residing in the B cell follicles, by sensing CXCL13, had no influence on follicular B cells number. The GC response formed upon immunization is dependent on chemokine sensing within the GC to migrate between the DZ, guided by CXCL12, and the LZ, guided by CXCL13 (23). Moreover, after affinity maturation in the GC, PC upregulate CXCR4 to migrate into the red pulp (24). B cells that lack CXCR4 fail to create clear LZ and DZ areas in GCs and PC fail to leave the B cell follicles (23–25). Upon immunization with TNP-SRBC, Rac1^B^Rac2^−/−^ mice had normal frequency of GC and PC, suggesting that the follicular Rac1^B^Rac2^−/−^ B cells *in vivo* could overcome the severely reduced migratory response *in vitro*. One possibility is that efficient T cell activation achieved by a strong TD antigen such as TNP-SRBC can overcome the B cell deficiency. In this context, it is interesting to note that in the absence of Cdc42, B cells mount an efficient response to TNP-SRBC, whereas the response to the soluble TD antigen NP-KLH is reduced (4). In humans, Rac2 seems to act dominantly over Rac1 for antibody production since loss of Rac2 or dominant negative Rac2 leads to common variable immunodeficiency with low antibody serum titers (26–28).

The elevated IgG2b response after *in vitro* activation of Rac-deficient B cells is puzzling The effect was most evident in the absence of both Rac1 and Rac2, but also Rac1^B^ and Rac2^−/−^ single deficient B cells and B cells expressing Rac2 from only one allele (Rac2^+/−^ B cells) showed increased IgG2b switching. Our results indicate that Rac1 and Rac2 or downstream signaling molecules suppress Ig class switching to IgG2b. Ig class switching is dependent on activation of GL transcripts covering the region to which switching will occur (17). Indeed, we found elevated GL γ2b levels in Rac1^B^Rac2^−/−^ activated B cells, when compared to WT B cells. This indicates that molecules downstream of Rac1 and Rac2 are involved in transcriptional control leading to Ig class switching. It is, to our knowledge, not known what type of transcription factors regulate GL γ2b transcription. A puzzling observation is that whereas IgG2b responses were highly elevated in Rac1^B^Rac2^−/−^ B cells, the IgG3 response was unaltered or sometimes lower. Both IgG2b and IgG3 switching are activated by LPS although the regulation on which of these two isotypes is selected remains unknown (29). Our previous observation is that the ratio IgG2b:IgG3 is very constant within individuals of one mouse strain, but may differ when comparing different strains. The ratio is also constant in the kinetics of Ig class switching *in vitro* (unpublished observation). On the other hand, expression of these two isotypes *in vivo* may differ a lot. Thus, IgG2b contributes substantially to the total IgG response to a TD stimulus, whereas IgG3 is mostly stimulated by TI antigens (30, 31). Our data suggest that signaling from Rac1 and Rac2 may be involved in the selection of IgG2b and IgG3 class switching. Engagement of promoters with distal enhancer elements in long-range looping interactions has been implicated in regulation of Ig class switch recombination (32). One possibility is that signaling molecules downstream of Rac1 and Rac2 activation directly participate in the induction of chromatin accessibility for GL γ2b transcripts.

Our data, as well as those presented by Henderson et al. (6), show that Rac2 is more important for B cell responses than Rac1. However, in many responses, the phenotype of the Rac1^B^Rac2^−/−^ mice is more severe than that of Rac2^−/−^ mice. This suggests that Rac1 can replace Rac2 to some extent. Since Rac1 is deleted only in B cells, the difference between the single and double knockouts cannot be explained by defects in other cell types than B cells and thus must be B cell intrinsic. We have in this paper showed that Rac1 and Rac2 are involved in many types of B cell responses and further investigations are necessary to fully understand the mechanism behind this. It is also evident that several signaling pathways might be involved in the different responses. Our study shows that Rac1 and Rac2 are involved in many B cell activities.

## Ethics Statement

All mouse strains were bred in specific pathogen-free conditions in the animal facility of the Department of Molecular Biosciences, the Wenner-Gren Institute, Stockholm University. All experiments using mice were approved by a local ethical committee on animal experiments.

## Author Contributions

NG, ES, and LW designed research and drafted the manuscript; NG, MH, CD, NK, and ES performed the research; NG, MH, CD, NK, ES, and LW analyzed the data and edited the manuscript.

## Conflict of Interest Statement

The authors declare that the research was conducted in the absence of any commercial or financial relationships that could be construed as a potential conflict of interest.
