# Supplementary figures and images for: The Small Rho GTPases Rac1 and Rac2 Are Important for T-Cell Independent Antigen Responses and for Suppressing Switching to IgG2b in Mice
Source: Front Immunol. 2017 Oct 6;8:1264. doi: 10.3389/fimmu.2017.01264 (PMC5635268; doi:10.3389/fimmu.2017.01264)

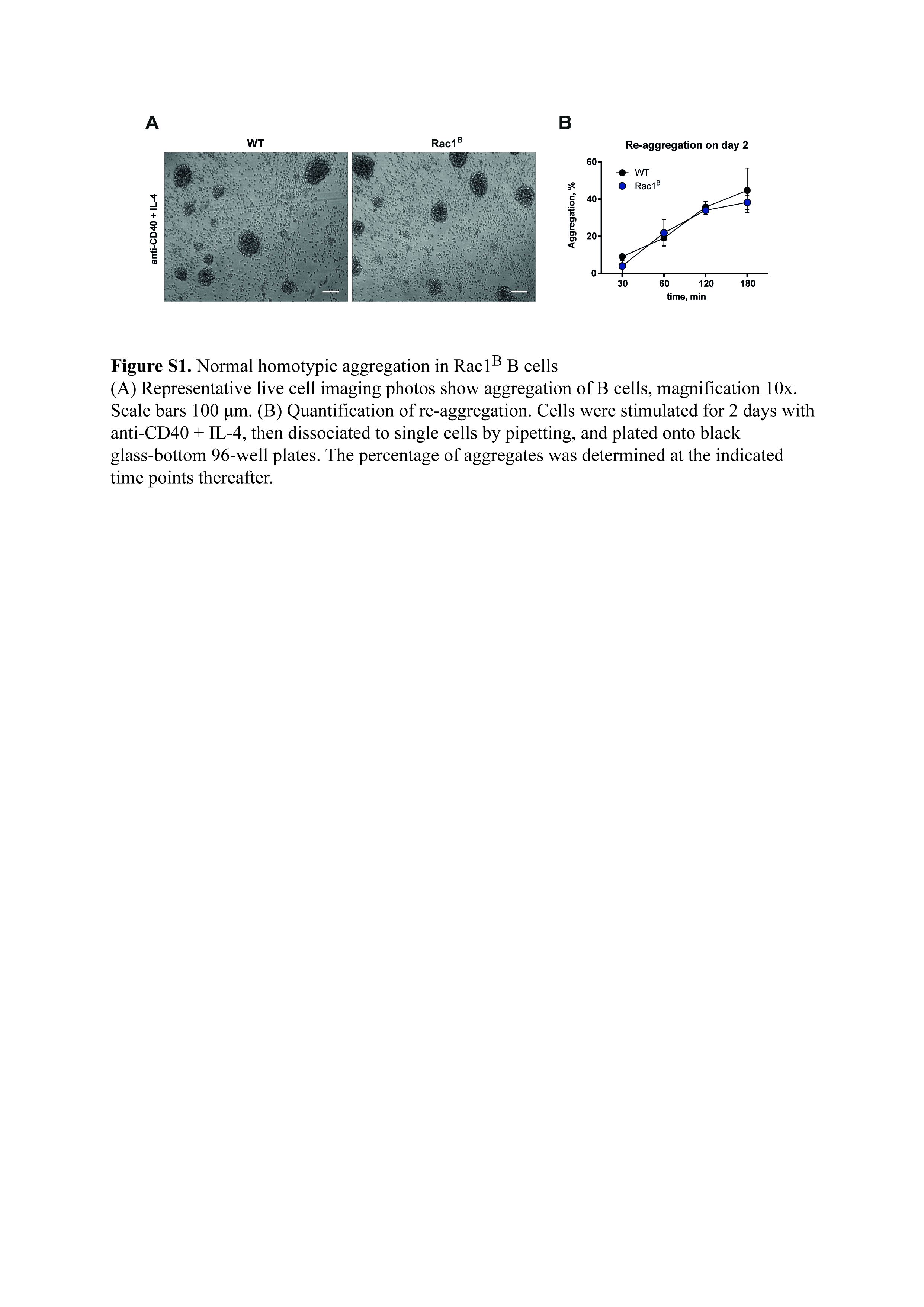

Supplement: Supplementary file 1 [file Image_1.TIF]

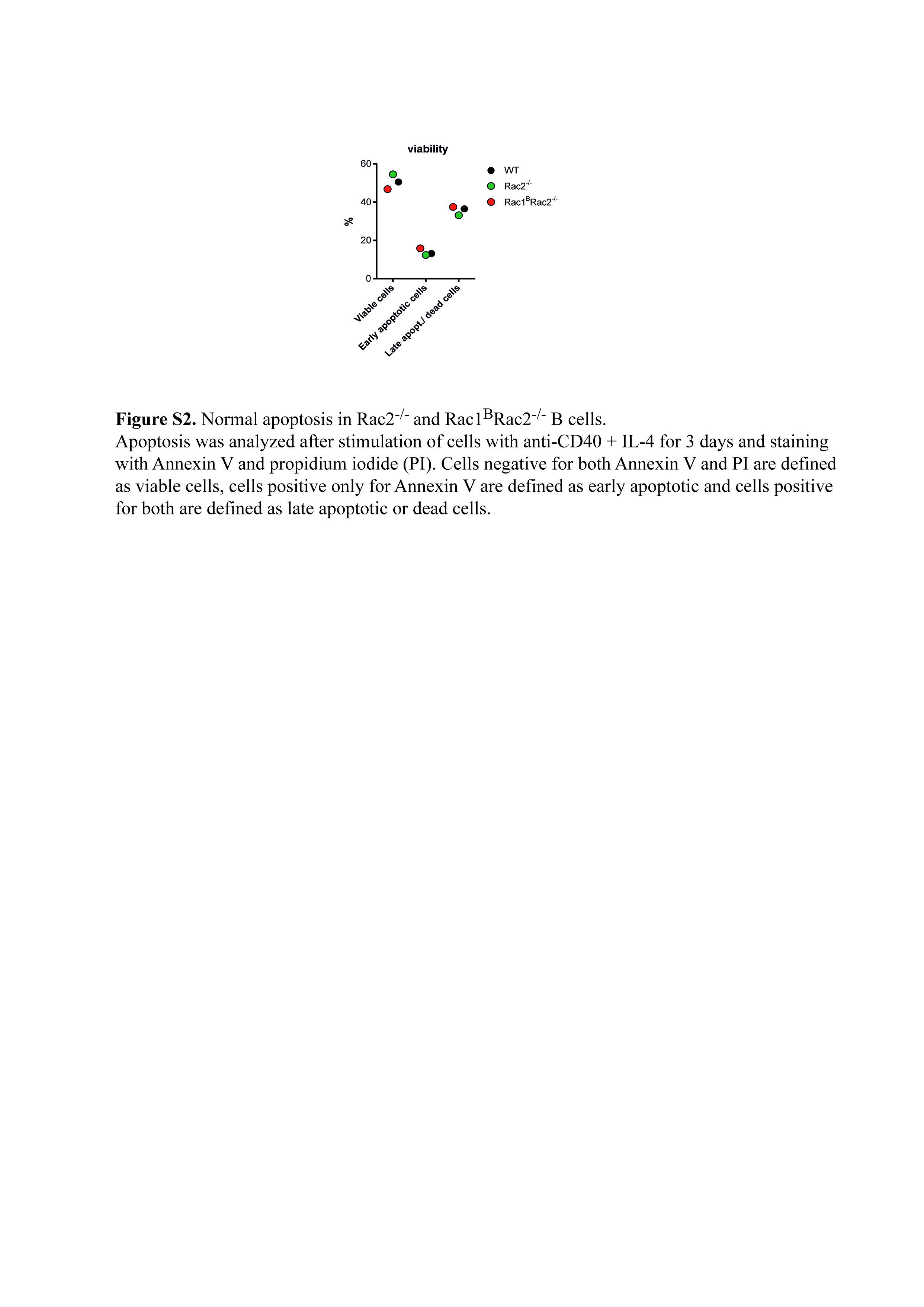

Supplement: Supplementary file 2 [file Image_2.TIF]
